# Us and them: identifying cyber hate on Twitter across multiple protected characteristics

**DOI:** 10.1140/epjds/s13688-016-0072-6

**Published:** 2016-03-23

**Authors:** Pete Burnap, Matthew L Williams

**Affiliations:** 1 Cardiff School of Computer Science & Informatics, Cardiff University, Cardiff, UK; 2 Cardiff School of Social Sciences, Cardiff University, Cardiff, UK

**Keywords:** cyber hate, hate speech, Twitter, NLP, machine learning

## Abstract

Hateful and antagonistic content published and propagated via the World Wide Web has the potential to cause harm and suffering on an individual basis, and lead to social tension and disorder beyond cyber space. Despite new legislation aimed at prosecuting those who misuse new forms of communication to post threatening, harassing, or grossly offensive language - or cyber hate - and the fact large social media companies have committed to protecting their users from harm, it goes largely unpunished due to difficulties in policing online public spaces. To support the automatic detection of cyber hate online, specifically on Twitter, we build multiple individual models to classify cyber hate for a range of protected characteristics including race, disability and sexual orientation. We use text parsing to extract typed dependencies, which represent syntactic and grammatical relationships between words, and are shown to capture ‘othering’ language - consistently improving machine classification for different types of cyber hate beyond the use of a Bag of Words and known hateful terms. Furthermore, we build a data-driven blended model of cyber hate to improve classification where more than one protected characteristic may be attacked (*e.g.* race and sexual orientation), contributing to the nascent study of intersectionality in hate crime.

## Introduction

The evolution of the World Wide Web from a static linked content publishing platform to a highly interactive real-time broadcast medium through which billions of people are able to publish their current thoughts, feelings and beliefs has revolutionised public communication. While the benefits of this are massive in terms of bringing people together and enabling distributed communities to be connected, one unanticipated drawback of this is the ability for hateful and antagonistic content - or cyber hate - to be published and propagated [1, 2]. Several studies have shown how individuals with prejudicial views towards a range of minority groups are taking to the Web to spread such hateful messages [3–5]. Oksanen *et al.* [6] reported 67 per cent of 15 to 18 year olds in a study of social media users had been exposed to cyber hate on Facebook and YouTube, with 21 per cent becoming victims of such material. Instances of cyber hate and racist tension on social media have also been shown to be triggered by antecedent events, such as terrorist acts [1, 2, 7]. This is a not only morally and ethically problematic. Recently, cyber hate has become a legal issue in many countries, and custodial sentences have been given to people who use the Web to spread and incite hatred based on individual characteristics such as race, religion and sexual orientation. Arguably the UK (England and Wales) is the most progressive in this area. Legislation pertaining to England and Wales that protects people from threatening, harassing, or grossly offensive speech online includes the Offences Against the Person Act 1861, the Public Order Act 1986, the Malicious Communications Act 1988, the Protection from Harassment Act 1997, and the Criminal Justice Act 2003. Similar laws also apply in France, Denmark and the Netherlands. In the US there are protections against posting harassing messages on the Web, without exposing personal identity.

In 2013, for first time, representatives from some of the leading social media companies came together with politicians and academics at a meeting of the Inter-parliamentary Coalition for Combating Anti-Semitism (ICCA) Task Force on Internet Hate at Stanford University. It was established that it is extremely difficult to respond to cyber hate due to scale, definition and classification [8]. The outcome of the meeting was to establish ‘Best Practices for Responding to Cyber Hate’ [9] that recommend timely and proportionate responses from social media providers, and for the Internet community to explore avenues for counter-speech as a viable alternative to criminal sanctions.

However, despite increasing evidence that cyber hate is on the rise, the availability of legislation to bring about prosecution, and the desire from leading social media companies to reduce harm, it goes largely unpunished given the multiple difficulties in policing online public spaces. Of these difficulties, classifying cyber hate in a timely manner, and at scale, are the most challenging given increasing restrictions on policing resources [10] and the difficulty with identifying appropriate opportunities to engage in counter speech. Therefore, automated techniques are needed that programmatically classify cyber hate to lighten the burden on those that have a responsibility to protect the public. This task is non-trivial given the number of ‘protected characteristics’, including race, religion, disability, sexual orientation and transgender status. Each characteristic is associated with specific hate related terms complicating the task of automated classification. The task is further complicated by the intersection of multiple identities in single victims. The debate on intersectionality in hate crime scholarship, while nascent, has begun to unpack how various identities interact and are read by victims and perpetrators. For example, a limited literature reporting on the intersectional nature of homophobic and transphobic (*e.g.* [11]), Islamophobic and genderphobic (*e.g.* [12]) and homophobic and racist (*e.g.* [13]) victimisation has begun to emerge. Intersectionality therefore presents a particular challenge for the automated identification of cyber hate.

Furthermore, gauging public ‘levels’ of cyber hate following major incidents is a key requirement for policing. More than half of all hate-related terrestrial attacks following 9/11 occurred within two weeks of the event [14]. It is during this period that policy and decision makers may require additional intelligence due to the lack of real-time insight into wide scale social reaction following an event based on reported crimes. Open source communications, such as social media data, lend themselves to this purpose given their inherent fine-grained temporal characteristics. Social media posts are produced by the second, while curated and administrative data have a much higher degree of latency in terms of both availability to decision makers and measurement of reaction. Thus, an automated cyber hate classification system could support more proactive public order management in the first two weeks following an event, and reduce harm to targeted social groups in an appropriate manner.

In this paper we built on previous work that developed a machine classification system to automatically detect religious cyber hate in Twitter posts [1]. We aimed to build a more generalisable model that could address the aforementioned challenge of intersectionality by providing evidence to support the hypothesis that classification can be improved by developing a blended model that incorporates knowledge of how different protected characteristics (*e.g.* race and sexuality) intersect in cyber hate speech. The contribution of the research is twofold. The primary contribution is a set of supervised machine classification models for multiple protected characteristics - race, disability and sexual orientation - to complement the existing classifier for religion. The systematic generation of features that support classification was applied across multiple cyber hate *types*, with consistent improvement in classification performance. A secondary contribution is an exploratory single blended model of cyber hate that incorporates knowledge of features across multiple types. In this instance, the blended model is shown to improve classification performance for instances of cyber hate in an intertextual context.

## Datasets

In this study, the aim was to build cyber hate speech classifiers for text that is targeted towards individuals or social groups based on their race, sexual orientation or disability. This builds on previous work that developed a machine classification system for religious cyber hate [1]. Transgender cyber hate was not considered as part of the study. As hate crimes have been shown to spike following antecedent or ‘trigger’ events [14], study data sets were collected from Twitter for a period immediately following selected ‘trigger’ events. Twitter was selected as the data source because it differs from other online social networks, such as Facebook and Google+, in that posts are largely public, programmatically accessible, and free to academic researchers. The open nature of Twitter also allows larger groups of people to interact publicly, something that is less common between individuals or small groups in other social networks. Twitter effectively supports a digital agora that promotes real-time interactive exchange of thoughts, opinions and beliefs, making it a defensible and well-suited source for data for this research. The selected ‘trigger’ events were: for race, the presidential re-election of Barack Obama starting November 6th 2012; for sexual orientation, the public announcement by Jason Collins on 30th April 2013 - the first active athlete in an American professional sports team to come out as gay. This dataset was specifically chosen because of its intersectional nature. Jason Collins is homosexual and black and thus likely to be targeted based on sexual orientation and race; and for disability, the opening ceremony of the Paralympic games in London, UK on 29th August 2012. Data collection used search terms based on named entities that were the focus of the event *i.e.* ‘obama’, ‘jason collins’, ‘paralympic’. These terms would include many references to the events and the main hashtags surrounding the event *e.g.* ‘# paralympics’. The hashtag convention is widely used on Twitter to link an individual’s thoughts and comments to an event. Data were collected for a two-week window following the start of an event. This specific duration was selected for two reasons. First, existing research indicates that public interest in events typically spikes a short time after the event, and then rapidly declines [15]. Second, as more than half of all hate-related attacks following 9/11 occurred within two weeks of the event [14], it is assumed that this time window would provide us with the widest variety, and the largest number, of hateful responses.

Building models to classify data according to a predefined coding scheme is an essential task in data science, especially in research involving machine classification of subjective matter. Building a model to predict house prices can use historical and factual data. Building a model to predict emotions, beliefs or sentiments (such as hateful remarks) in electronic text requires an additional step to establish a ‘gold standard’ that is suitable for training and testing supervised machine classifiers, and is based on human agreement on which class a piece of text belongs to. Commonly, this is obtained by sampling from a larger data set and employing human annotators to label each data point (tweet) according to a coding frame ([16, 17]). The coding frame serves as a set of categories or classes into which each data point can be classified. Computationally crowdsourcing human annotations is now becoming popular, and Web services such as CrowdFlower or the Amazon Mechanical Turk provide programmatic application programming interfaces (APIs) through which researchers can automatically upload a data set, coding frame, and set of instructions for annotation. The results of the annotation tasks can then be split into training and testing data sets for machine learning.

Each event produced datasets between 300,000 and 1.2 million, from which we randomly sampled 2,000 to be human coded. Coders were provided with each tweet and the question: ‘is this text offensive or antagonistic in terms of race ethnicity/sexual orientation/disability?’ They were presented with a ternary set of classes - yes, no, undecided. We utilized the CrowdFlower online service that allows for Human Intelligence Tasks, such as coding text into classes, to be distributed over multiple workers. Workers can sign up to the service to participate in tasks in return for micropayments (small payments set by the task creator based on the number of tasks completed to an acceptable standard). Task creators can also specify a range of worker requirements such as location and experience, and can verify the level of expertise via test questions. Results from workers can then either be accepted or rejected, based on level of agreement with other workers.

CrowdFlower recruits from its pool of workers until each unit of analysis (in this case, each tweet) is annotated by a minimum number of workers, as specified by the task creator. We required at least four human annotations per tweet as per the convention in related research [18]. CrowdFlower provides an agreement score for each annotated unit, which is based on the majority vote of the trusted workers [19]. Because CrowdFlower continues to recruit workers until the task is complete, there is no guarantee that all workers will annotate the same set of units. Therefore we cannot calculate traditional inter-rater reliability scores, such as Krippendorf’s Alpha or Cohen’s Kappa to determine agreement between all annotators. However, CrowdFlower has been shown to produce an agreement score that compares well to these classic measures [19]. Based on the output from our annotator task we can determine agreement on each unit. The purpose of the experiments performed in this article are to establish the accuracy of a machine classifier when annotating tweets as hateful and/or antagonistic or not, and thus it is the agreement score for the unit of analysis (each tweet), and not the overall human agreement for all units that is important for validation. We removed all tweets with less than 75 percent agreement and also those upon which the coders could reach an absolute decision (*i.e.*, the ‘undecided’ class) - again, following established methods from related research [20]. The results of the annotation exercise produced three ‘gold standard’ data sets as follows: Sexual Orientation - 1,803 tweets, with 183 instances of offensive or antagonistic content (10.15% of the annotated sample); Race - 1,876 tweets, with 70 instances of offensive or antagonistic content (3.73% of the annotated sample); Disability - 1,914 tweets, with 51 instances of offensive or antagonistic content (2.66% of the annotated sample). The proportion of instances of offensive or antagonistic content, which we refer to after this point as cyber hate, is small relative to the size of the sample. However, these are random samples of the full datasets for each event and are therefore representative of the overall levels of cyber hate within the corpus of tweets.

## Automatically identifying cyber hate speech

Greevy & Smeaton [21] classified racist content in Web pages using a supervised machine learning approach with a bag-of-words (BOW) as features. A BOW approach uses words within a corpus as predictive features and ignores word sequence as well as any syntactic or semantic content. This approach can lead to misclassification due to word use in different contexts and, if words are used as a primary features for classification, it has been shown that combining sequential words into *n*-grams (list of words occurring in sequence from $1-n$) improves classifier performance by incorporating some degree of context into the features [22]. However, an *n*-gram approach can suffer from the problem of high levels of distance between related words - for example, if related words appear near the start and near the end of a sentence [23]. Dadvar, Trieschnigg, and de Jong [24] used profane words in a social media account username, references to profanities and bullying-sensitive topics, and first and second person pronouns to classify antagonistic behaviour on YouTube. Dinakar *et al.* [25] also focused on the identification of cyberbullying using a BOW approach, but also incorporated lists of profane words, parts-of-speech and words with negative connotations as machine learning features. Furthermore, they included a common-sense reasoning approach to classification by using a database that encoded particular knowledge about bullying situations (*e.g.*, associating wearing dresses with males).

Burnap *et al.* [17] developed a rule-based approach to classifying antagonistic content on Twitter and, similarly to [25], they used associational terms as features. They also included accusational and attributional terms targeted at a person or persons following a socially disruptive event as features, in an effort to capture the context of the term use. Their results demonstrated an improvement on standard learning techniques (see also [16]). Chen *et al.* [23] identified offensive content by using profanities, obscenities, and pejorative terms as features, weighted accordingly based on the associated strength of the term, as well as references to people. They also produced a set of rules to model offensive content, showing an improvement on standard machine learning approaches in terms of a much-reduced false negative rate.

**Figure 1:**
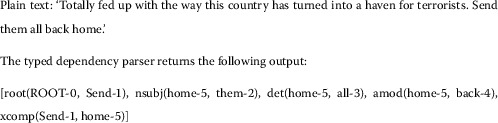
**Example of text transformation to typed dependency feature set.**

Burnap *et al.* [1] identified that ‘othering’ language was a useful feature for classifying cyber hate based on religious beliefs - specifically for identifying anti-muslim sentiment. Othering is an established construct in rhetorical narrative surrounding hate speech [26], and the ‘we-they’ dichotomy has long been identified in racist discourse [27]. Examples of language that distanced particular social groups geographically (*e.g.* ‘send them home’), attempted to justify an expectation of malicious behaviour from the group (*e.g.* ‘told you so’), and was openly derogatory (*e.g.* ‘muslim savages’) were reported on Twitter following the murder of Lee Rigby by Islamist extremists in London, 2013 [1]. Following the effectiveness of identifying othering terms and their success as features in a machine classifier for cyber hate targeted at specific religious groups, the present research aimed to test the effectiveness of the ‘us and them’ model on other types of hate speech to develop evidence for the generalizability of this method. To extract potential othering terms the Stanford Lexical Parser was implemented, along with a context-free lexical parsing model, to extract typed dependencies within the tweet text [28]. Typed dependencies provide a representation of syntactic grammatical relationships in a sentence (or tweet in this case) that can be used as features for classification, and have the potential to capture othering language. The following example explains the meaning of such relationships and how they can be used as features to inform the machine classifier. Consider the sentence in Figure 1.

Within the output we can see five instances of typed dependencies. The second instance (nsubj(home-5, them-2)) identifies a relationship between ‘home’ and ‘them’, with ‘home’ being the fifth word in the sentence and ‘them’ appearing before ‘home’ as the second word. Word order within a sentence is preserved in the type dependency and provides a feature for classification as well as the syntactic relationship between words. The relationship identified by the parser in this case is nsubj, which is an abbreviation of nominal subject. This will include a noun phrase (‘them’), which is the syntactic subject in the sentence, and an associated relational term (‘home’). Linguistically therefore, the term ‘them’ is associated with ‘home’ in a relational sense. Sociologically, this is an othering phrase, which essentially distances ‘them’ from ‘us’ through the relational action of removing ‘them’ to their ‘home’, as perceived by the author of the tweet. Similarly, the third typed dependency (det(home-5, all-3)) identifies a det relationship, which is short for determiner, where a link is established between a noun phrase and its determiner. The noun phrase here being ‘home’ (as in a place) and the determiner being ‘all’. Again, this falls into an othering behaviour, suggesting that the entire social group should have a relationship with ‘home’, which we can assume means the perceived ‘home’ of the social group by the author of the tweet (*i.e.*, ‘not my country’). For further explanation of the other features there is a complete documentation in [28]. This combination of linguistics and sociology potentially provides a very interesting set of features for the more nuanced classification of cyber hate, beyond the BOW approach that utilizes expletives and derogatory terms. It allows a more common-sense reasoning approach to classifying cyber hate by considering the integration of othering terms and calls for retribution action into the classification features.

## Feature preparation and modelling

The first set of features used was a Bag of Words (BOW). For each tweet the words were stemmed using the Snowball method and transformed to lowercase before being split into *n* grams of size 1-5, retaining 2,000 features, with word frequency normalised for each vector. The second feature was extracted by identifying known hateful terms and phrases for hate speech based on race, disability and sexual orientation. These were extracted from a crowd-sourced list of terms on Wikipedia.^1^ ^2^ ^3^ The final set of features were the typed dependencies. Each tweet was transformed into typed dependency representation using the Stanford Lexical Parser, transformed to lowercase, and split into *n* grams of size 1-3, retaining 2,000 features, with frequency normalisation applied.

Machine classification experimentation was performed using (i) a Support Vector Machine (SVM) algorithm with a linear kernel, and (ii) a Random Forest Decision Tree algorithm with 100 trees. The rationale for the selection of these methods is based on previous research that analysed the performance of a range of alternative methods using similar data to those used in this study, and reported that these methods produced optimum results [1]. It was evident for the experiments performed in the present research that SVM continually outperformed the Random Forest approach, as such only the SVM results are reported for brevity. Experiments were also conducted using RBF and Polynomial kernels using SVM to establish whether a non-linear model fitted the data better, but both of these produced models with very poor detection of cyber hate. The SVM parameters were set to normalize data and use a gamma of 0.1 and C of 1.0, refined through experimentation.

## Results

### Individual models of cyber hate speech

The first set of results document the findings of applying machine classification to cyber hate directed towards each protected characteristic individually, based on disability, race, and sexual orientation. Religion is also included in the results for comparison, based on previous research using typed dependencies to detect a single type of cyber hate [1]. The results shown are for cyber hate detection rates only. The classification performance for non-hate text was consistently above 0.95-0.98 and are omitted to reduce complexity in presenting the results. Our main interest is with the improvement of cyber hate classification.

For this set of results a 10-fold cross-validation approach was used to train and test the supervised machine learning method. This approach has previously been used for building machine classifiers for short text [18]. It functions by iteratively training the classifier with features from 90 percent of the manually coded data set, and classifying the remaining 10 percent as ‘unseen’ data, based on the features evident in the cases it has encountered in the training data. It then determines the accuracy of the classification process and moves on to the next iteration, finally calculating the overall accuracy.

**Figure 2:**
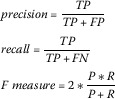
**Formula for generating performance metrics.**

The results of the classification experiments are provided using standard text classification measures of: precision (*i.e.*, for class *x*, how often are tweets classified as *x* when they should not be (false positives) - a measure of true positives normalised by the sum of true and false positives); recall (*i.e.*, for class *x*, how often are tweets not classified as *x* when they should be (false negatives) - a measure of true positives normalised by the sum of true positives and false negatives); and *F*-Measure, a harmonized mean of precision and recall. The results for each measure range between 0 (worst) and 1 (best). The formulae for calculating these results are shown in Figure 2 (where TP = true positives, FP = false positives, TN = true negative, and FN = false negative).

The aim of the research was to produce a system to identify instances of cyber hate posted to online social networks such as Twitter. Thus, one objective was to identify the features that reduce false negatives - so as to minimise instances of cyber hate missed. A second objective was to reduce false positives - so the system minimises instances of ‘false alarms’. The experiments conducted produce metrics that can be compared to determine the optimum individual features or combinations. As such, number of false positives and false negatives for each feature/type (of hate speech) couple are provided in the results. These are not intended to be compared between types of hate speech (horizontally), but between feature sets (vertically). Table 1 provides machine classification performance results for four different protected characteristics. Religion is provided as a baseline from previous research, and disability, race and sexual orientation are new results. The key finding from previous research was that the inclusion of typed dependencies in the classification of religious cyber hate reduced the false negative rate by 7% when compared to using hateful terms alone [1, 2]. From the new results we can infer the following insights: For disability, there is no significant improvement in using typed dependencies over a standard BOW approach. However, the hateful terms provided no contribution to the classification of this type of hate speech. Using hateful terms alone results in everything being classified as non-hate.

**Table 1:** **Machine classification performance for cyber hate based on disability, race and sexual orientation (results rounded to 2dp)**

	**Religion (baseline)**			**Disability**			**Race**			**Sexual orientation**		
	**P**	**R**	**F**	**P**	**R**	**F**	**P**	**R**	**F**	**P**	**R**	**F**
*n*-Gram words 1 to 5 with 2,000 features	0.80 FP = 38	0.69 FN = 69	0.74	0.969 FP = 1	0.608 FN = 20	0.73	0.72 FP = 15	0.54 FN = 32	0.62	0.53 FP = 67	0.42 FN = 107	0.47
*n*-Gram hateful terms	0.89 FP = 19	0.66 FN = 75	0.76	0.00	0.00	0.00	0.93 FP = 3	0.53 FN = 33	0.67	1.00 FP = 0	0.098 FN = 165	0.18
*n*-Gram words (1-5) with 2,000 features + hateful terms	0.74 FP = 58	0.65 FN = 78	0.69	0.89 FP = 4	0.61 FN = 20	0.72	0.79 FP = 13	0.71 FP = 20	0.75	0.57 FP = 60	0.44 FN = 105	0.49
*n*-Gram typed dependencies	0.53 FP = 48	0.24 FP = 168	0.33	0.97 FP = 1	0.61 FP = 20	0.75	0.87 FP = 3	0.29 FN = 50	0.43	0.95 FP = 2	0.22 FN = 142	0.36
*n*-Gram typed dependencies+hateful terms	0.89 FP = 19	0.69 FN = 70	0.77	0.97 FP = 1	0.61 FP = 20	0.75	0.91 FP = 4	0.59 FN = 29	0.71	0.96 FP = 2	0.27 FN = 134	0.42
*n*-Gram words (1-5) with 2,000 features + *n*-Gram typed dependencies+hateful terms	0.89 FP = 19	0.69 FN = 70	0.77	0.97 FP = 1	0.61 FN = 20	0.75	0.87 FP = 7	0.66 FN = 24	0.75	0.72 FP = 25	0.35 FN = 119	0.47

For race, there is a 13% reduction in false negatives when including typed dependencies as an additional feature together with hateful terms, while retaining performance in the false positive rate. Typed dependencies also provide a 10% reduction in false negatives over a BOW model, while reducing the false positive rate by almost 4*x*. This is a significant improvement for the classification of racial cyber hate and suggests the typed dependency inclusion is necessary for improving classifier performance. The lowest false negative rate is achieved with by combining the BOW and hateful terms - but the lack of typed dependencies in this model leads to a higher false positive rate. Overall, the best performance for racial cyber hate is achieved by blending the BOW, hateful terms and typed dependency features sets. This combination returns a very low false positive rate, and a false negative rate reduction of 38% over the use of hateful terms alone.

Classification results for sexual orientation exhibit similar characteristics to the race results in that: (i) hateful terms alone yield very poor performance; (ii) the combination of BOW and hateful terms produces the lowest false positive rate, and (iii) introducing typed dependency features has a significant improvement on the false positive rate - reducing it by up to 30*x* - but to the detriment of false negative performance, which will lead to missed instances of hate speech. Overall, the highest *f*-measure for sexual orientation was achieved by combining BOW and hateful terms. This model produced a 28% improvement over the combination of all three features sets. However, the importance of typed dependencies remains evident by producing nearly 2.5*x* fewer false positives in the combined model, leading to an *f* measure of only 0.02 below that of BOW and hateful terms.

To summarise this experiment, we have produced evidence to suggest the inclusion of typed dependencies as features in the classification of cyber hate reduced false positive rate in the classification of 2 out of 3 types of hate speech - race and sexual orientation - when compared to using a BOW model and/or hateful terms as features. Typed dependencies combined with BOW and hateful terms also produced overall classification performance results that were equal to, or better than BOW and hateful terms alone in 2 out of 3 types of hate speech - race and sexual orientation.

### Blended models of cyber hate speech

The first experimental phase produced evidence to suggest that including probabilistic syntactic and grammatical language features in a predictive model of cyber hate speech in short informal text, such as Twitter posts, will improve performance. The second phase of this research was to determine the possibility of developing a more generalizable model of cyber hate. The motivation for this was to explore the potential for building a model that is capable of handling intersectional cyber hate where more than one protected characteristic is targeted. To do this we followed a number of data-driven experiments to establish the effectiveness of ‘cross-pollination’ between samples of individual types of cyber hate, by mixing samples at the training stage.

First, to determine the effectiveness of each individual model in classifying cyber hate for other protected characteristics, we cross-validated across all classes on an individual basis - training on one and testing on another (results shown in Table 2). Second, to determine the effectiveness of mixing instances of cyber hate across protected characteristics in improving classification of individual types of hate speech, we drew a random sample from each individual dataset (race, sexuality and disability) and combined the samples into a single dataset for training. We retained the same proportions of hate/non-hate as in the individual datasets so as not to artificially improve performance by altering the balance of classes in training. Two experiments were conducted using this data. One aimed to determine the effectiveness of the blended model in improving cyber hate classification on a binary basis (hate/non-hate) (results shown in Table 3). For the other, we relabeled the training data to retain not only the cyber hate label but also the protected characteristic to which the hate speech was directed (race-hate, race-non-hate, sexual-orientation-hate etc.). This experiment aimed to establish improvements in detecting individual types of cyber hate when combining features. Theoretically, this was motivated by the observation that there may be some use of multiple types of hateful language when the context of the remark includes individuals or groups that have intersectional protected characteristics - for example, Jason Collins is homosexual and black. The results of a data-driven experiment are necessary to measure any improvement in classifying the sexual orientation dataset - which would then suggest the combination of models based on context, rather than a single model of cyber hate.

**Table 2:** **Cross validation of different types of cyber hate**

	**Training data**									
		**Disability**			**Race**			**Sexual orientation**		
		**P**	**R**	**F**	**P**	**R**	**F**	**P**	**R**	**F**
Testing Data	Disability	0.96 FP = 1	0.61 FN = 20	0.75	0.00 FP = 0	0.00 FN = 51	0.00	0.00 FP = 1	0.00 FN = 51	0.00
	Race	0.00 FP = 1	0.00 FN = 70	0.00	0.87 FP = 7	0.64 FN = 25	0.74	0.95 FP = 1	0.29 FN = 50	0.44
	Sexual orientation	0.00 FP = 2	0.00 FN = 183	0.00	1.00 FP = 0	0.09 FN = 165	0.18	0.74 FP = 23	0.37 FN = 116	0.49

**Table 3:** **Binary cyber hate classification using a combined dataset of 3 different protected characteristics**

	**P**	**R**	**F**
Non-hate	0.97	0.99	0.98
Hate	0.79 (FP = 62)	0.59 (FN = 162)	0.68
Overall	0.96	0.97	0.96

Table 2 illustrates very clearly that individual models of cyber hate do not generalise well across different protected characteristics. In all but two cases, the trained models did not detect any cyber hate in test instances from a different protected characteristic. However, one case where there was an improvement was when the model was trained using homophobic cyber hate instances and tested using the race dataset. This suggests that there were features present within the sexual orientation data that were relevant to racism. In this case, it is possible that the sexual orientation training data also contained racist narrative due to the context of the case. The homophobic hate speech was directed at a black male. There is a smaller reciprocal improvement in classification performance when using sexual orientation as the test dataset after having trained the model on racist cyber hate. In this case, the racist element of the sexual orientation dataset is likely being predictive of racist cyber hate, but to a lesser degree. These results suggest people posting this content were targeting more racist content towards a black homosexual man than they were targeting homophobic remarks to a black heterosexual man. This presents an interesting future research direction - determining whether it is possible to measure the likelihood of attacking more than one protected characteristic with varying degrees of frequency in certain cases, to dynamically improve classification of hate speech following a new event. If this were measurable, it may be possible to mix training datasets on-the-fly and rebuild supervised classification models to reflect the context of the antecedent event and measure public reaction in real-time. For example, if a terrorist attack was carried out by a member of a minority group in a predominantly caucasian community in the UK/US, it would be expected that there may follow a hateful response. If the individual or group responsible for the attack exhibited particular race characteristics different to caucasian, the response would be expected to reflect on that. If it transpired later that they also exhibited or supported religious or sexuality beliefs, the response may also reflect on that. As the context changed, it would be likely that the cyber hate classifier would also require updating to maintain levels of accuracy.

The dynamic production of context-specific training data is beyond the scope of this study, but to provide some evidence for the utility in producing blended models of cyber hate, and measuring their relative improvement on individual models, Table 3 shows the results of an experiment where all individual datasets - race, disability and sexual orientation - are combined into a single dataset, and used to train and test a model using the same SVM configuration as the earlier experiments and the combination of BOW, hateful terms and typed dependency features. The model is tested using 10-fold cross validation. The mixed dataset contained 6,486 tweets, with 6,091 containing no cyber hate and 395 containing cyber hate. The mean precision of the individual classifiers for cyber hate was 0.85, the mean recall 0.54, and the mean *f*-measure 0.656. The combination of individual training data into a single model reduced mean precision to 0.79 but improved recall to 0.59 and *f*-measure to 0.68, suggesting that ‘cross-pollination’ of training data actually improves the performance of cyber hate classification across all classes - most likely by capturing intersectional hate speech.

To better understand how this is improving the classification of cyber hate for individual protected characteristics, Table 4 shows the performance for the individual classes using the same combined single dataset but retaining the separate class labels. From this we see a slight drop in precision for each type when compared to the individual models - 0.97 to 0.91 for disability, 0.87 to 0.86 for race, and 0.72 to 0.66 for sexual orientation. For recall - disability remains unchanged, race drops from 0.66 to 0.60, but sexual orientation improves from 0.35 to 0.41. As per the cross validation results in Table 2, this supports the possibility that blending datasets where the context of the cyber hate could contain multiple types of hate speech due to intersectionality (in this case, homophobic and racist) will improve classification results. It is encouraging here to note that while Table 1 reports results for sexual orientation hate speech with P = 0.57, R = 0.44 and F = 0.49 when using a single classifier, Table 4 reports sexual orientation hate speech results of P = 0.66, R = 0.41 and F = 0.51. There is a small drop in recall, which the confusion matrix (see Table 5) showed was due to classification as non-hate, rather than confusing sexual orientation hate speech with other classes. Despite the small drop in recall, there is a significant increase in precision due to a 62% decrease in false positives when being exposed to features from other types of hate speech. The blended results offer supporting evidence that exposing a supervised machine learned model to different types of hate speech can improve results if the training data can suitably blended to capture an intersectional context. However, this must be carefully constructed because it appears the inclusion of training data from alternative protected characteristics can cause confusion within the supervised classification model and lead to a drop in precision performance. Table 5 shows classifier output with expected class on the vertical axis and machine classification result on the horizontal. Ideally, numbers greater than zero would be in the diagonal cells that cut through these, and every other cell would be 0. Reflecting on Table 5, some confusion appears between the non-hate classification based on race and disability, but generally misclassification is contained to the individual classes, with confusion between hate and non-hate. This suggests there remains some latent features within the text that require further exploration to continue this line of research.

**Table 4:** **Multi-class cyber hate classification using a combined dataset of 3 different protected characteristics**

	**P**	**R**	**F**
Non-hate-disability	0.95	0.97	0.96
Hate-disability	0.91	0.61	0.73
Non-hate-race	0.95	0.96	0.95
Hate-race	0.86	0.60	0.71
Non-hate-sexual orientation	0.94	0.97	0.95
Hate-sexual orientation	0.66	0.41	0.51

**Table 5:** **Confusion matrix for multi-class cyber hate classification using a combined dataset of 3 different protected characteristics**

**a**	**b**	**c**	**d**	**e**	**f**	**←** **classified as**
1,798	3	61	0	1	0	a = non-hate-disability
18	31	2	0	0	0	b = hate-disability
74	0	1,724	7	0	1	c = non-hate-race
0	0	28	42	0	0	d = hate-race
3	0	3	0	1,577	37	e = non-hate-sexual orientation
0	0	0	0	108	75	f = hate-sexual orientation

### Example typed dependencies from blended model

In Table 6 we present some of the most highly weighted features from the blended model. That is, features that contribute highly to the classification of each type of cyber hate. The interpretation of these is somewhat subjective but given the narrow context of the events, and the fact they are highly predictive of text labelled as cyber hate by human annotators, we can make some assumptions about the meaning of these terms. For sexual orientation and race types we can see that ‘othering’ terms continue to be present. References to ‘the closet’, ‘absolute disgrace’ and ‘kill yourself’ are all used in a derogatory and separatist manner, intended to denigrate and offend based on sexual orientation. Similarly, ‘is destroying’, ‘white people’ and ‘won…black’ are used in racist cyber hate to differentiate white from black people in the context of the event, perhaps even suggesting skin colour had some outcome on the election. For disability cyber hate we can see less explicit ‘othering’ terms and more of a focus on mocking disabled athletes using terms such as ‘jokes’, ‘really drunk’ and ‘wish…falling’. In all three cases we can see why using typed dependencies has improved the classification outcome by identifying features that a BOW or hateful terms model would not identify, and incorporating co-occurring but often distant terms (2 or 3 words apart with different terms inbetween).

**Table 6:** **Confusion matrix for multi-class cyber hate classification using a combined dataset of 3 different protected characteristics**

**Typed dependency**	**Explanation**
*Homophobic samples*	
det(backdoor-7, the-6)	Determiner (a specific reference to a noun phrase) discussing ‘the backdoor’ in a context of homosexual activity
dobj(kill-2, yourself-3)	Direct object (an accusatory object of the verb) suggesting homosexual ‘others’ should ‘kill yourself’
det(closet-8, the-7)	Determiner (a specific reference to a noun phrase) discussing ‘the closet’ - most likely referring to where the person should have remained
amod(disgrace-6, absolute-5)	Adjectival modifier (a descriptive phrase related to a noun phrase) discussing ‘disgrace’ - and amplifying this accusation with ‘absolute’
det(disgrace-6, an-4) aux(commending-12, him-13)	Determiner (a specific reference to a noun phrase) discussing ‘disgrace’ - plus Auxiliary ‘commending’, branding people commending the person a disgrace
*Race samples*	
advcl(won-7, black-11) advcl(won-7, obama-13)	Two adverbial clause modifiers relating ‘won’ and ‘obama’ & ‘won’ and ‘black’ - highlighting the colour of skin as a key related term to the victory
aux(destroying-10, is-9)	Auxiliary verb potentially suggesting Obama is having a ‘destroying’ impact
amod(people-7, white-6), advmod(won-11, how-9)	Modifiers linking ‘white people’ to the outcome of the election outcomes ‘how…won’
dobj(see-13, you-14)	Direct object (an accusatory object of the verb) referring to ‘you’ and the impact the outcome may have
*Disability samples*	
amod(athletes-11, olympic-10) advmod(drunk-14, really-13)	Modifiers referring to ‘olympic athletes’ and ‘really drunk’ in mocking manner referring to ‘you’ and the impact the outcome may have
det(jokes-10,the-9)	Referring to noun ‘joke’ in relation to paralympic athletes
amod(women-12,disabled-11) dobj(falling-13,wish-15)	Modifier of ‘women’ to refer to ‘disabled’ female athletes and ‘wish’ they would be ‘falling’ using direct object
amod(bench-11, midget-9)	The key term here being the derogatory term ‘midget’

## Conclusions

In this paper we developed novel machine classification models to identify different types of cyber hate individually and intersectionally. We used text parsing to extract typed dependencies, which represent syntactic and grammatical relationships between words, and are shown to capture ‘othering’ language - consistently improving machine classification for different types of cyber hate beyond the use of a Bag of Words and known hateful terms, which have been the main method for identifying cyber hate previously.

Furthermore, we built a data-driven blended model of cyber hate to improve classification where more than one protected characteristic may be attacked (*e.g.* race and sexual orientation), contributing to the nascent study of intersectionality in hate crime. The results for this model suggest that if a context can be established for the likely factors that may trigger hateful responses, a bespoke supervised model could be built using a blend of historical training data relating to these factors.

The extraction of typed dependencies that were most predictive of each class label within the blended model identified co-occurring terms and exact type of cyber hate language - highlighting ‘othering’ terms for sexual orientation and race, and mockery in the language of hate speech targeting disability.

Some limitations remain to be addressed in future research. First, while typed dependency examples improved classification in the majority of the supervised models, they also illustrated how specific predictive terms are related to event-related contexts. Thus we need more cases to expand the model to be more generalisable. This is particularly relevant for cases with an intersectional dimension. Future research should seek cases where religion intersects with sexual orientation, or race intersects with disability and sexual orientation etc. Second, the cases in this research focused on western examples in UK/US. Cases should also be selected from different world regions and cultures. Future studies should consider cases in non-western cultures where tolerance toward minority characteristics may be different. Finally, the results of the models indicate room for improvement in the identification of features - identifying novel ways to measure latent hateful and antagonistic meaning within the language of cyber hate. One direction could be to investigate interaction between users as well as classifying tweets in isolation.

The resulting cyber hate classification models have been shown to be applicable to a range of protected characteristics including race, disability and sexual orientation, and provide new ability to automatically identify content perceived by a group of human annotators as hateful or antagonistic. Instead of requiring a human moderator to observe and monitor online social networks for such content in the aftermath of potential ‘trigger’ events, our approach will help inform those responsible for managing such content, and allow them to verify and react, rather than have to search for offensive content in large streams of data.

## Footnotes

1
https://en.wikipedia.org/wiki/List_of_ethnic_slurs.

2
https://en.wikipedia.org/wiki/List_of_LGBT_slang_terms.

3
https://en.wikipedia.org/wiki/List_of_disability-related_terms_with_negative_connotations.
